# A Glimpse Into the Structure and Function of Atypical Type I Chaperonins

**DOI:** 10.3389/fmolb.2018.00031

**Published:** 2018-04-11

**Authors:** Mohammed Y. Ansari, Shekhar C. Mande

**Affiliations:** National Centre for Cell Science, Pune, India

**Keywords:** Type I chaperonins, GroEL, GroES, *Mycobacterium tuberculosis*, protein folding, gene duplication

## Abstract

Chaperonins are a subclass of molecular chaperones that assist cellular proteins to fold and assemble into their native shape. Much work has been done on Type I chaperonins, which has elucidated their elegant mechanism. Some debate remains about the details in these mechanisms, but nonetheless the roles of these in helping protein folding have been understood in great depth. In this review we discuss the known functions of atypical Type I chaperonins, highlighting evolutionary aspects that might lead chaperonins to perform alternate functions.

## Molecular chaperones

Molecular chaperones comprise of a wide range of proteins playing key roles in cellular homeostasis and are responsible for assisting in protein folding, assembly of multimeric proteins, translocation of proteins within and across cell, degradation of unwanted, or misfolded proteins during normal cellular processes and stabilization of proteins by preventing aggregation and assisting in refolding under stress conditions (Lindquist, 1986; Lindquist and Craig, 1988).

Proteins reported to have chaperone activity were initially discovered as those overexpressed during heat shock and hence were named as the heat shock proteins (Hsp). Apart from heat shock, other stress condition such as carbon, nitrogen, or phosphate limiting conditions were also known to induce molecular chaperones. These proteins are classified according to their molecular weight into five major families: (a) Hsp100 family, (b) Hsp90 family, (c) Hsp70 family, (d) Hsp60 family, and (e) small heat shock protein family (sHsp) (Bohen et al., 1995; Schirmer et al., 1996; Bukau and Horwich, 1998). The chaperones are also classified based on their mode of action into: (a) Foldases, Chaperones that assist refolding of unfolded proteins by using ATP, e.g., Hsp70 and Hsp60, (b) Holdases, Chaperones that bind folding intermediates and prevent aggregation, e.g., sHsp and Hsp40, and (c) Disaggregases, Chaperones which actively disaggregate the harmful protein aggregates, which might lead to their small fragments, e.g., members of AAA + ATPase superfamily and Hsp100. This type of classification holds true with few exceptions (Richter et al., 2010; Kim et al., 2013). Much of our understanding on the mechanisms of chaperone-assisted protein folding has been derived from work on Hsp60 and Hsp70 families of chaperones. This review focuses on Hsp60 class of molecular chaperones, highlighting Hsp60 with atypical structure and function.

### Hsp60 family/chaperonins

**Figure 1:**
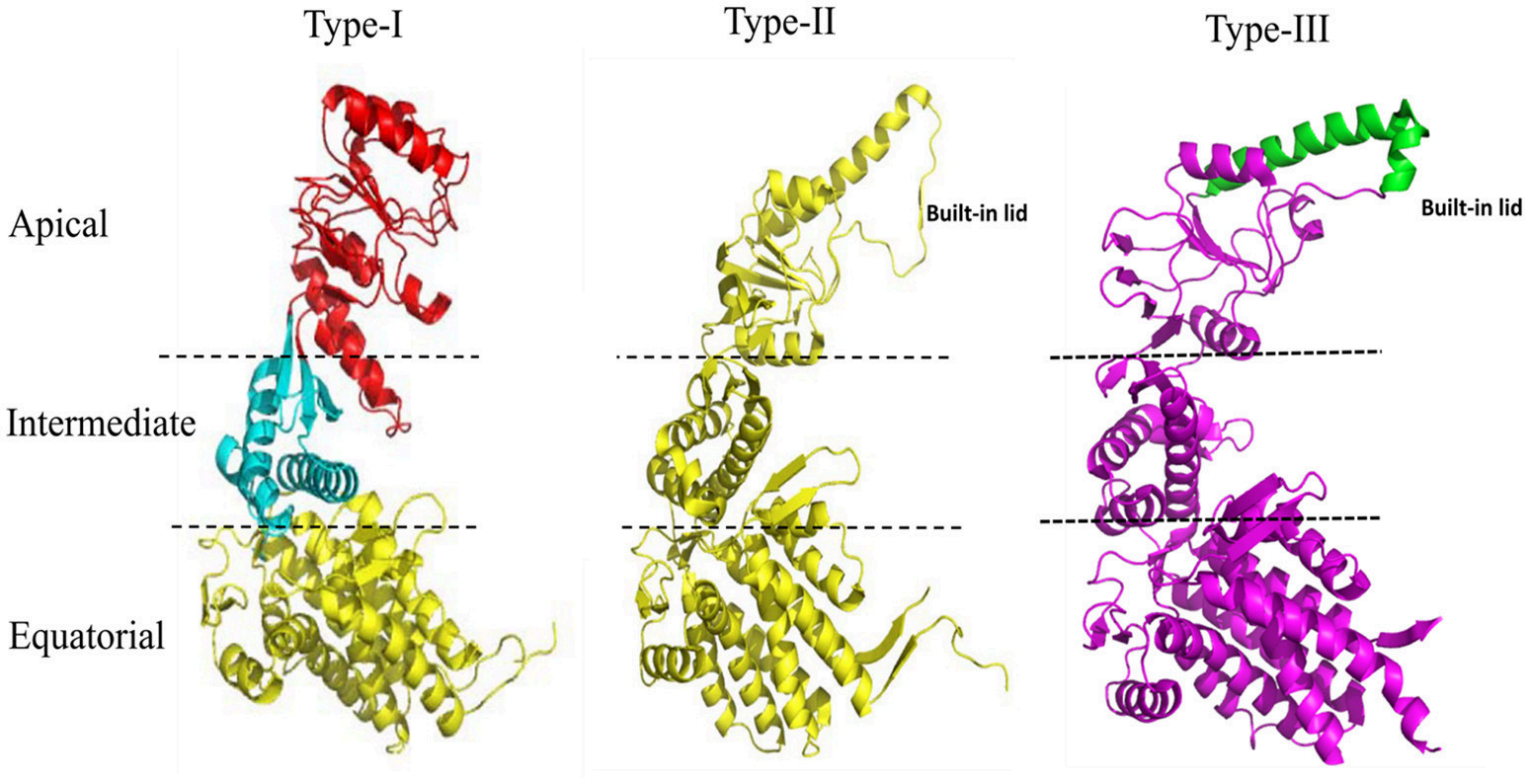
Structural features of the Type I, Type II, and Type III chaperonins. The comparative structure analysis of Type I, Type II, and Type III chaperonins. Structures were downloaded from the RCSB with codes of PDB: 1AON, 3RUW, and 5X9U, respectively. Type I chaperonin is demarcated into Apical, Intermediate, and Equatorial domains, analogous regions of which are shown in Type II and III chaperonins using dotted lines. Type II chaperonin has a characteristic built-in lid in the structure that plays the role of co-chaperonin GroES of Type I chaperonin. Type III chaperonins are structurally similar to Type II chaperonins in having built-in-lid. However, the sequence, structure and function of the lid are distinct in Type II and Type III chaperonins (An et al., 2017). The PyMOL program (PyMOL Molecular Graphics System, version 1.3) was used to generate this figure.

The 60 kDa chaperones form large oligomeric rings, and are also referred to as the chaperonins. Chaperonins can be further sub-classified into two groups on the basis of requirement of co-chaperonins and their cellular location. Type I chaperonins are found in the cytoplasm of prokaryotes and in the mitochondrion and chloroplast of eukaryotes. They require the assistance of the co-chaperonin i.e., Hsp10, which acts as a cap on the ring. The well-studied Type I chaperonin is known as the GroEL-GroES system in *Escherichia coli*. Its homologs are Cpn60/Cpn20 in chloroplasts, and mtHsp60/mtHsp10 in mitochondrion (Cheng et al., 1989; Hayer-Hartl et al., 1995; Dickson et al., 2000). Type II chaperonins are found in the cytoplasm of eukaryotes and in the archaebacterial micro-organisms. They have an in-built lid and hence do not require co-chaperonins for their function (Ranson et al., 1998). Example of Type II chaperonin includes eukaryotic TriC/CCT machinery (TCP-1 ring complex/chaperonin containing TCP-1 complex), which is made up of 8 subunits and the thermosome in archaebacteria. Contrary to Type I chaperonins, substrate independent capture of Type II chaperonins require the assistance of prefoldin and Hsp70 homologs (Iizuka et al., 2004; Cuéllar et al., 2008). Recently, a third group known as Type III chaperonins was reported which are structurally similar to Type II chaperonins but mechanistically and phylogenetically distinct from both Type I and Type II chaperonins e.g., *Carboxydothermus hydrogenoformans* chaperonin (Ch-CPN) (Techtmann and Robb, 2010; An et al., 2017; Figure 1). The Type I, II, and III chaperonins are also known as Group I, II, and III chaperonins.

### Structure-function of Type I chaperonins: prokaryotic cytosol

#### *E. coli* GroEL-GroES

Structural and functional studies on *E. coli* GroEL have shown that it forms a tetradecameric structure composed of two heptameric rings stacked on each other forming a cavity, which changes its character from being predominantly hydrophobic to hydrophilic upon binding GroES. Substrate protein folding takes place in this cavity with the assistance of co-chaperonin GroES, which is a cap-like heptameric structure (Mande et al., 1996). Each GroES monomer is of 10 kDa size. The GroEL monomer is demarcated into three domains namely apical, intermediate, and equatorial domain. Each monomer is ~57 kDa in size.

There are two models proposed for the GroEL-GroES mediated substrate protein folding. Asymmetric/sequential model, which is accepted widely. In this model the GroEL and GroES are present stoichiometrically in 2:1 ratio (14:7 subunit ratio). In the other model known as the symmetric/simultaneous model, which is based on the recently observed GroEL-GroES complex, both rings of GroEL are capped by co-chaperonin GroES in the stoichiometric ratio of 1:1 i.e., (GroEL-GroES)_2_, and subunit ratio of 14:14 (Sameshima et al., 2008; Ye and Lorimer, 2013; Fei et al., 2014). Symmetric (GroEL-GroES)_2_ complex has been observed both in the presence and absence of substrate protein suggesting a transient intermediate state in the folding reaction cycle.

### Structure-function of Type I chaperonins: endosymbiotic organelles

#### Chloroplast and mitochondrial chaperonins

The chloroplast chaperonins are typically referred to as Cpn60 (GroEL homologs) and Cpn10 (GroES homologs). The Cpn60 chaperonins are made up of multiple subunits which are diverged into two related but distinct α and β types (Dickson et al., 2000; Hill and Hemmingsen, 2001). Contrary to bacterial chaperonins, which contain multiple subunits and prefer homo-oligomerization (Ojha et al., 2005; Gould et al., 2007), chloroplast chaperonins form hetero-oligomers with its two types of chaperonin α and β subunits. Heterogeneity also exists in the co-chaperonin structure. Cpn10 is similar to the standard co-chaperonin, forming heptameric single ring of 10 kDa subunits (Koumoto et al., 2001; Sharkia et al., 2003). Cpn20 has two Cpn10-like polypeptide sequences joined in tandem. The purified Cpn20 exists as a tetramer ring-like structure containing 20 kDa subunit. It is fully functional *in vitro*, helping refolding of denatured protein in presence of both chloroplast Cpn60 and *E. coli* GroEL (Tang et al., 2006). Moreover, the *Chlamydomonas reinhardtii* Cpn10 assist GroEL only in presence of Cpn20 (Tsai et al., 2012). Thus, a considerable heterogeneity exists in the oligomeric assembly of chloroplast chaperonins.

The human mitochondrial chaperonin, mtHsp60 is known to have a protein-folding mechanism (mitochondrial protein) distinct from GroEL-GroES system and requires a single heptameric ring to carry out its protein folding function along with its co-chaperonin, mtHsp10 (Viitanen et al., 1992; Nielsen and Cowan, 1998). However, the crystal structure of mitochondrial chaperonin in complex with its co-chaperonin, mtHsp60-mtHsp10 depicts a unique intermediate stage where mtHsp60-mtHsp10 forms a symmetric double-ring football-like structure, (mtHsp60)_14_ + 2 (mtHsp10)_7_.

### Type I chaperonins: non-canonical features

#### Multiple chaperonins across species

Analysis of completely sequenced genomes suggest that about 30% of all the genomic sequence data possess multiple copies of gene sequences encoding chaperonins (Lund, 2009; Kumar et al., 2015). Distribution of these multiple chaperonins based on extensive phylogenetic analysis suggest that multiple copies of chaperonin genes exist predominantly in five phyla, namely, (a) phylum *Actinobacteria*, (b) phylum *Firmicutes*, (c) phylum *Cyanobacteria*, (d) phylum *Chlamydia*, and (e) α*-Proteobacteria* phylum (Kumar et al., 2015).

##### Actinobacteria

*Actinobacteria* are Gram-positive bacteria and possess high G + C content in their genomes, e.g., *Mycobacterium tuberculosis, Mycobacterium leprae*, and *Bifidobacterium longum*. These species typically possess two copies of chaperonin genes, with one of the copies being present on an operon-like structure. The other copy of Cpn60 exists as an independent gene without the presence of Cpn10 gene (Rinke de Wit et al., 1992). The actinobacterial chaperonin genes are under the regulatory control of HrcA transcription factor which binds to upstream CIRCE (controlling inverted repeat of chaperone expression) sequence (Duchêne et al., 1994; Grandvalet et al., 1998). In some cases regulation is mediated through HspR transcription factor binding to upstream HAIR (HspR Associated Inverted Repeat) sequence (Barreiro et al., 2004).

##### Firmicutes

*Firmicutes* are Gram-positive bacteria and possess low G + C content in their genomes, e.g., *Staphylococcus aureus, Desulfitobacterium dehalogenans*, and *C. hydrogenoformans*. *Firmicutes* are known to possess both prokaryotic-like Type I chaperonin genes and archael-like chaperonin genes classified as Type III chaperonin. Type I chaperonins are arranged in an operonic arrangement with the co-chaperonin, while Type III chaperonin gene is located in the *dnaK* operon. Both the Type 1 and Type III chaperonin genes are regulated by HrcA transcription factor (Techtmann and Robb, 2010).

##### Chlamydiae

*Chlamydia*e are mostly obligate intracellular pathogens, e.g., *Chlamydia trachomatis, Chlamydia pneumonia*, and *Chlamydia psittaci*. Chlamydial species possess three copies of chaperonin genes (McNally and Fares, 2007). Operonic arrangement suggests that only one copy of the chaperonin genes exists along with its co-chaperonin. However, other chaperonin genes are located separately. Regulation of chlamydial chaperonin genes is complex. The first copy of the chaperonin gene is induced by heat shock and regulated by HrcA-CIRCE system. The second copy of the chaperonin gene is induced when *Chlamydia* are inside monocyte or macrophages (Kol et al., 1999), and the third copy of the chaperonin gene is induced when *Chlamydia* are in Hep-2 cells (Gérard et al., 2004). Such types of expression and regulation of chaperonin genes suggest life-cycle specific patterns and independent functional roles for them.

##### α-proteobacteria

*Rhizobia*, which belong to the α*-proteobacteria* class, are symbiotic organisms living in association with leguminous plants in the root nodules and are involved in nitrogen fixation, e.g., *Bradyrhizobium japonicum, Rhizobium leguminosarum*. *Rhizobia* contain most number of copies of chaperonins. *B. japonicum* has seven copies of chaperonin genes (Fischer et al., 1993). *R. leguminosarum* is a well-characterized organism and has three copies of chaperonin genes. Gene arrangement in all these organisms suggests that the three copies of the chaperonin gene form separate operons with their respective co-chaperonin genes (George et al., 2004). One of the chaperonin operons is located on the genomic island that contains genes involved in nitrogen fixation. It is regulated by NiF factors that regulate nitrogen fixation genes (Ogawa and Long, 1995). The second copy of the chaperonin gene is not well-studied and is known to be involved in chaperoning property of several model substrate proteins (George et al., 2004).

##### Cyanobacteria

*Cyanobacteria* are largely photosynthetic bacteria, e.g., *Synechococcus platensis, Prochlorococcus marinus*, and *Anabaena variabilis*. About 90% of the genomic sequences of the cyanobacterial species contain two copies of chaperonin genes with one of them being arranged on an operon while the other chaperonin gene coded separately. Some cyanobacterial species containing three copies of chaperonin genes, where two of its chaperonin genes being located with respective co-chaperonins in the operon while the third copy of chaperonin genes is independent (Lund, 2009; Kumar et al., 2015). Chaperonin genes existing in the operonic arrangement with their co-chaperonins are essential genes while the ones which exist independent of the co-chaperonin are non-essential (Sato et al., 2008). The two cyanobacterial chaperonin genes are positively regulated by RpoH and negatively regulated by HrcA proteins. Upon heat shock, one of the chaperonin genes is induced rapidly while the other chaperonin gene is gradually induced (Kojima and Nakamoto, 2007; Rajaram and Apte, 2010). The chaperonin gene that is gradually induced on heat shock is known to be directly involved in photosynthesis.

### Evolutionary lineage

As more genomic sequences are becoming available, analysis of chaperonin genes suggests that distribution and frequency of multiple copies of chaperonin genes across phyla and organisms continues to increase (Lund, 2009; Kumar et al., 2015). In order to understand the cause of multiplicity of chaperonin genes is either due to horizontal gene transfer or gene duplication, phylogenetic analysis was carried on GroEL proteins across species, which revealed that the causes of existence of multiple copies of GroELs are non-uniform. In a few cases there is gene duplication event followed by evolutionary selection such as that observed in myxobacterial GroELs, mycobacterial first and second copy of GroEL and few rhizobial GroELs. In the case of the third mycobacterial GroEL homolog, few rhizobial GroELs and methanosarcinal GroELs, horizontal gene transfer occurred (Goyal et al., 2006; Kumar et al., 2015).

**Figure 2:**
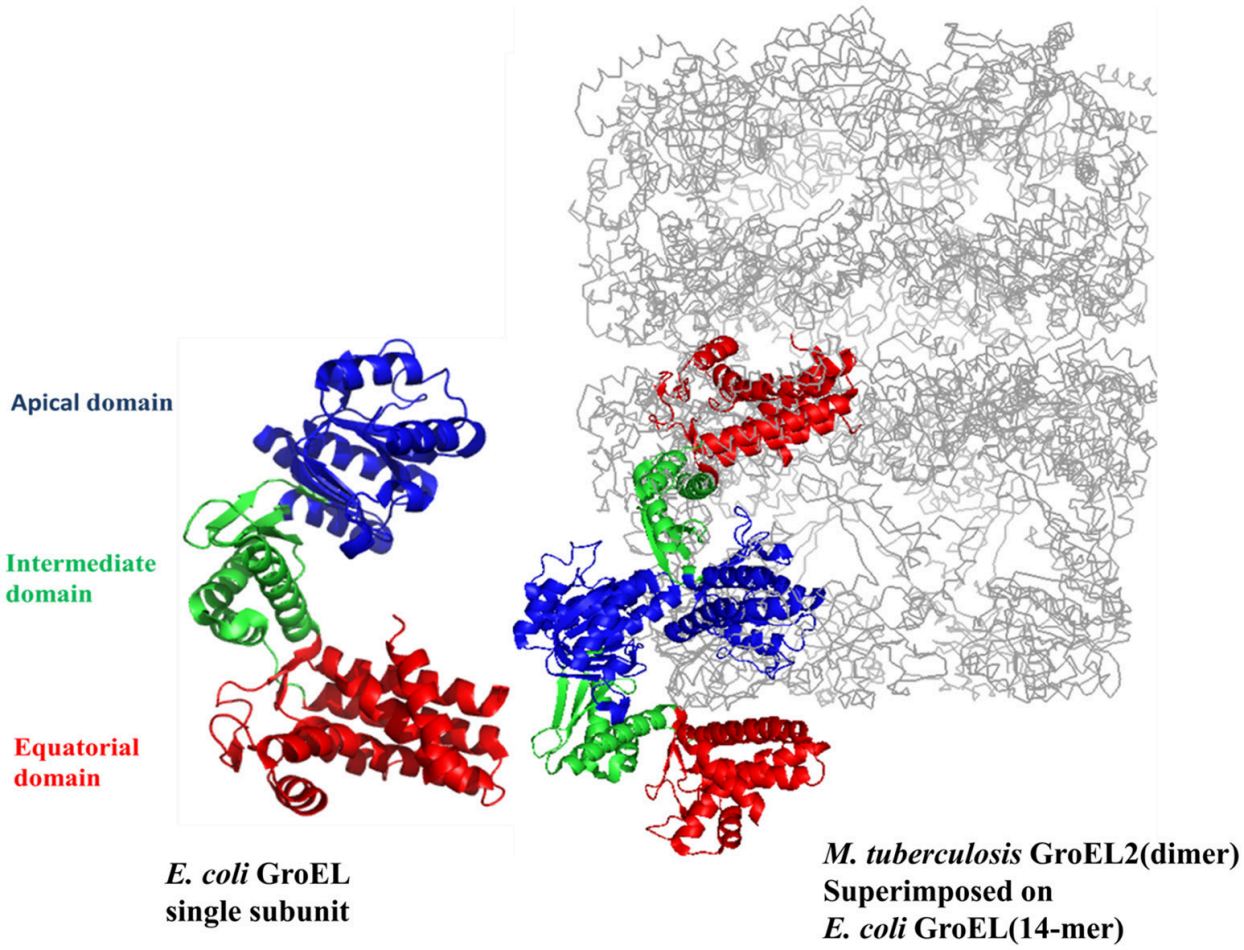
The crystal structure of *M. tuberculosis* GroEL2 superimposed on *E. coli* GroEL-ES structure. The structure of *M. tuberculosis* GroEL2 (PDB ID:1SJP) shows lower oligomeric status (dimer). Colored in blue, green, and red are the Apical, Intermediate and Equatorial domain, respectively. Compared to *E. coli* GroEL (PDB ID: 1AON) shown in gray color, the inter-subunit interaction is mediated through Apical domain in *M. tuberculosis* GroEL2 structure whereas inter-subunit interaction is through Equatorial domain in *E. coli* GroEL. Single subunit of *M. tuberculosis* GroEL2 is aligned to *E. coli* GroES bound GroEL ring representing asymmetric model. GroES structure has been removed for simplicity. A single subunit of *E. coli* GroEL has been shown with the same color-coded domains compared to *M. tuberculosis* GroEL2 for comparative analysis. The PyMOL program (PyMOL Molecular Graphics System, version 1.3) was used to generate this figure.

It has been proposed earlier in our lab that mycobacterial GroEL has been duplicated and undergone various selective pressures to perform distinctive structural and functional role during the course of evolution (Goyal et al., 2006). Biophysical and biochemical studies on recombinantly purified *M. tuberculosis* GroELs have shown that GroEL1 and GroEL2 exist as lower oligomeric species contrary to tetradecameric GroEL structure of *E. coli* (Qamra et al., 2004). The crystal structure of *M. tuberculosis* GroEL2 in its dimeric form highlighted the presence of distinct residues at the interface region, probably responsible for the change in oligomerization (Figure 2; Qamra and Mande, 2004). Gene shuffling and domain swapping studies on *M. tuberculosis* GroEL1 suggest that the equatorial domain is responsible for failed oligomerization. The apical domain can withstand large insertions and deletions (Kumar et al., 2009). Around the same time it was shown that GroEL1 has evolved to promiscuously bind nucleic acids (Basu et al., 2009) and oligomerization is facilitated by phosphorylation of serine residues (Kumar et al., 2009). Since GroEL2 is known to be essential chaperonin in *Mycobacteria*, whereas the oligomeric assembly of GroEL1 is regulated post-translationally, it was reported that tetradecameric assembly and precise inter-domain communication are prerequisite for chaperonin activity (Chilukoti et al., 2015).

### Functional diversity

It is important to examine whether the presence of multiple copies of chaperonins are responsible for behaving as canonical chaperonins or they have diverged to carry out novel functions. It is also important to note whether these multiple chaperonins act on common substrates or on distinct pool of substrates. GroELs are highly conserved across different species and it has been shown that homologs of chaperonins from other bacteria are able to function in *E. coli* suggesting overlapping of substrate proteins and common mechanism of GroEL function. The interactions of substrate proteins with GroEL are hydrophobic in nature, so conformational change mediated exposure of the apical and the equatorial domains in the cavity plays a key role in substrate recognition and assists protein folding. Binding of substrate proteins to GroEL is through α/β domains of proteins with no sequence similarity (Kerner et al., 2005; Kumar and Mande, 2011) and further studies suggest that GroEL selectively binds globular substrates rather than extended polypeptides (Robinson et al., 1994; Goldberg et al., 1997). Multiple copies of chaperonins in an organism have also been reported to have evolved to carry out novel functions. GroEL homolog in an insect symbiont, *Xenorhabdus nematophila* has been shown to be toxic to insects which is mediated through binding to alpha-chitin. Mutational analysis on these GroEL homologs suggests that the amino acid critical for this kind of activity is distinct from the essential chaperonin (Joshi et al., 2008). In *M. tuberculosis*, GroEL2 acts as a generalist chaperonin (Hu et al., 2008) while GroEL1 is reported to be associated with nucleoids (Basu et al., 2009). Thus, it is apparent that gene duplication of *groEL* genes has led to the functional diversity of chaperonins and/or distinct substrate spectrum for intracellular protein folding.

### Post-translational modifications/biofilm formation

Post-translational modifications in proteins are employed by organisms to modulate their physiological processes and adapt to constantly changing environment (Bernal et al., 2014). Chaperonins have been reported to be post-translationally modified in certain organisms, and this modification has been reported to gain/loss of their function. For example, fractionation of *M. tuberculosis* cell lysate has shown that tetradecameric form of GroEL1 is attained only upon phosphorylation at serine residues (Kumar et al., 2009). Similarly in another report it has been shown that phosphorylation occurs at threonine residues (Canova et al., 2009). Both of these observations suggest that oligomerization of GroEL1 is a result of post-translational modification.

Many pathogens evade innate immune response and become resistant to antibiotics by forming biofilms on epithelial cells (Hall-Stoodley and Stoodley, 2005). The role of GroEL in biofilm formation has been elucidated in a few organisms. For example, GroEL1 mutant of *M. smegmatis* fails to form biofilm. Mechanistic studies revealed that *M. smegmatis* GroEL1 interacts with the KasA enzyme, which is critical for mycolic acid biosynthesis involved in biofilm formation (Ojha et al., 2005). Interestingly, it has been recently reported that GroEL in pathogenic strain *B. anthracis* gets phosphorylated and thereby modulates biofilm formation. These findings highlight that phosphorylation of GroEL has functional implications (Arora et al., 2017). Acetylation is another post-translational modification associated with *E. coli* and *M. tuberculosis* chaperonins, however a functional role has not yet been ascribed to this modification (Liu et al., 2014). Similarly, mitochondrial co-chaperonin (mtHsp10) undergoes acetyl modification and controls folding of mitochondrial proteins under excess nutrient condition (Lu et al., 2015).

### C-terminal diversity

Various studies highlight the importance of the C-terminal residues of GroEL in the overall functioning of the chaperonin (Tang et al., 2006; Chen et al., 2013). In cases pertaining to multiple copies of chaperonins, they have distinct pattern of C-terminal residues. While the C-terminus of GroEL (from *E. coli*) has a 13 residue motif (GGM)_4_M, GroEL homologs from other organisms (which contain multiple copies of chaperonins) have distinct C-terminal motifs, such as:

a) Histidine-rich C-terminal, e.g., *Mycobacteria* (Colaco and MacDougall, 2014)

b) Pattern-less C-terminus, e.g., *Rhizobia* (George et al., 2004)

c) Similar (GGM)_4_M repeats, e.g., *Myxobacteria* (Wang et al., 2013)

d) Lack of GGM-like tail, e.g., *Methanosarcina* (Figueiredo et al., 2004)

**Figure 3:**
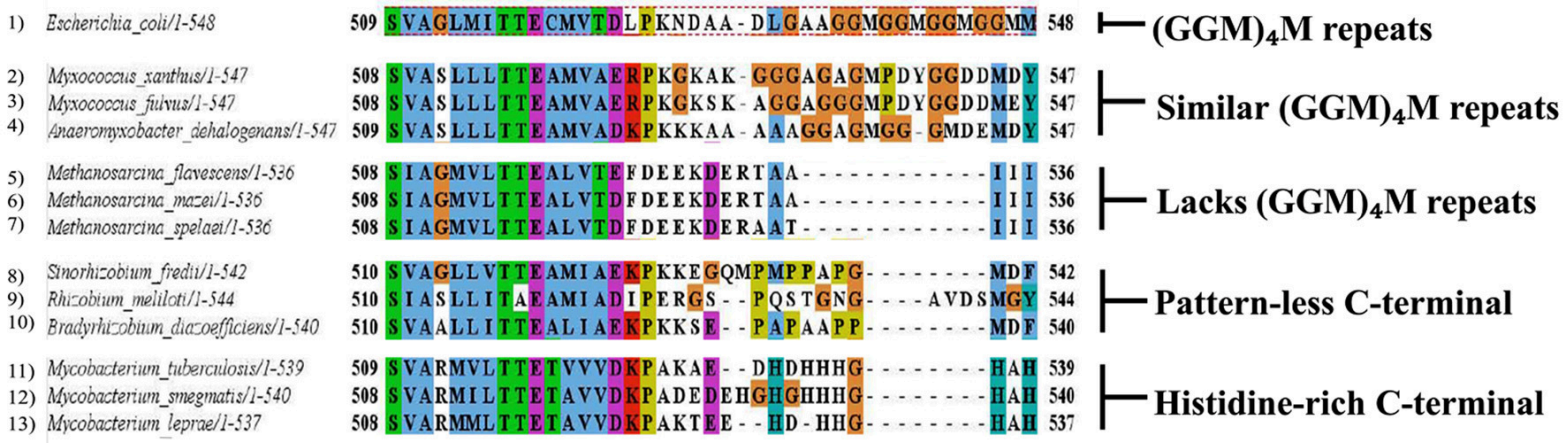
Multiple chaperonins in bacteria displaying diversity at C-terminal. Sequence alignment highlighting C-terminal regions of the representative bacterial GroEL homologs with the *E. coli* GroEL. The last C-terminal residues of selected multiple GroELs in different bacteria show divergence from the canonical (GGM)4M motif of the *E. coli* GroEL shown in dotted red box. Sequences were retrieved from www.uniprot.org and aligned in MEGA6 using MUSCLE algorithm (www.megasoftware.net). Formatting of aligned sequences were done in Jalview alignment viewer (www.jalview.org). Residues in the alignment follow the default Clustal color scheme of Jalview.

It is clearly seen that many chaperonin paralogs in different organisms have GGM-like C-terminus. A wide range of genomic organization is seen in these chaperonins. Moreover, differences are also seen in their co-expression with co-chaperonin and essentiality of their function. Thus, these paralogs are perplexingly observed to be either essential or non-essential, co-expressed with their co-chaperonin or not co-expressed, and possibly function as housekeeping chaperonins. On the other hand chaperonins not possessing the GGM-like C-terminus have possibly evolved to carry out novel functions (Ojha et al., 2005; Wang et al., 2013; Figure 3).

## Concluding remarks

Type I chaperonins are important by virtue of their role in intracellular protein folding. GroEL-GroES system in bacteria helps folding of about 10–15% of cytosolic proteins. Various structures of GroEL solved in apo-form, nucleotide-bound form as well as in complex with co-chaperonin GroES attempt to explain the role of these chaperonins in protein folding (Saibil et al., 2013). The existence of multiple chaperonins and their role in varied functions hints evolutionary pressure toward adapting to different environmental conditions. The structure of *M. tuberculosis* GroEL2 highlights lower oligomeric state and more exposed hydrophobic surfaces, probably to increase substrate pool and energy conservation (Qamra and Mande, 2004; Qamra et al., 2004; Kumar and Mande, 2011). Owing to the presence of Histidine-rich C-terminal in multiple chaperonins, these have been proposed to help in alternate biological functions. *M. smegmatis* GroEL1 binding to iron may help in biofilm formation (Ojha et al., 2005). Survival defect of M. tuberculosis *groEL1* knockout strain under low aeration condition might help in oxygen sensing by directly binding to metals or help certain metalloproteins in folding (Sharma et al., 2016). The structure of other homologous chaperonin proteins will probably answer the myriad of questions associated with the novel functions of chaperonin homologs.

## Author contributions

All authors listed have made a substantial, direct, and intellectual contribution to the work, and approved it for publication.

### Conflict of interest statement

The authors declare that the research was conducted in the absence of any commercial or financial relationships that could be construed as a potential conflict of interest.
